# A Focal Attention-Based Large Convolutional Kernel Network for Anomaly Detection of Coated Fuel Particles

**DOI:** 10.3390/s25113330

**Published:** 2025-05-26

**Authors:** Zhaochuan Hu, Jiang Yu, Hang Zhang, Jian Liu, Ning Chen, Rong Li

**Affiliations:** 1 State Key Laboratory of Advanced Design and Manufacture for Vehicle Body, Hunan University, Changsha 410082, China; 2 College of Automotive and Mechanical Engineering, Changsha University of Science and Technology, Changsha 410114, China

**Keywords:** coated fuel particles, image classification, focal attention, large convolutional kernel

## Abstract

The coating thickness of fuel particles is a critical parameter for ensuring the safe operation of high-temperature gas-cooled reactors. However, existing detection technologies still face limitations in measurement accuracy, efficiency, and automation. Notably, accurate thickness measurement relies on the precise identification of anomalous particles, which is hindered by several key challenges. First, incomplete particles in edge regions introduce significant interference. Second, some anomalies exhibit weak morphological features, making them difficult to detect. To address these issues, this study proposes an innovative focal attention-based large convolutional kernel network detection framework comprising three core modules. First, a Vision Transformer backbone incorporating a Large Selective Kernel Module dynamically adapts multi-scale receptive fields to enable coordinated global and local feature perception. Second, the Multi-Scale Feature Fusion Module establishes cross-layer feature interactions to enhance responses to subtle anomalies. Third, the Focal Attention Module employs a dynamic convolutional attention mechanism to strengthen the saliency representation of critical regions. Experimental results demonstrate the effectiveness of the proposed method, reducing the false detection rate and miss detection rate of anomaly detection to 1.96% and 1.9%, respectively.

## 1. Introduction

Nuclear energy is an efficient and clean energy, and it plays a crucial role in addressing the current energy crisis [1,2]. However, it also faces challenges related to safety concerns and social acceptance. High-temperature gas-cooled reactors (HTGRs), as one of the most advanced fourth-generation nuclear technologies, offer inherent safety features, modular design, multifunctional applications, and high-temperature characteristics. The inherent safety largely depends on the design and quality of the fuel particle coatings [3,4,5]. These coated fuel particles, which are the fuel components of HTGRs, consist of a central fuel kernel and four coating layers [5,6]. These coating layers serve as the primary barrier to prevent the release of fission products, and their performance directly affects the safe operation of the nuclear reactor [5,6]. The thickness of the coating layers is a key factor influencing the performance of the particles. After particle fabrication, the coating thickness must be measured, and the results are used to adjust and improve the production process.

Our previous research [7,8,9], based on ceramographic methods [10,11] and machine vision techniques, led to the development of a visual inspection system for coating thickness measurement. This system enables fully automated and highly efficient thickness measurement. It first scans the entire metallographic sample under a low-magnification objective to acquire the coordinates of all particles. Then, it switches to a high-magnification objective for sequential image acquisition and analysis. However, during image analysis, we found that not all collected images were suitable for measurement due to issues such as surface contamination, image defocus, and coating layer fractures. These anomalies often result from improper sample preparation or storage. Analyzing such images could introduce significant deviations from actual values, thereby reducing accuracy. Therefore, anomaly detection before image analysis is essential.

The anomaly detection task in this system is essentially an image classification task, aiming to categorize different images into distinct classes while minimizing classification errors. Traditional methods primarily rely on handcrafted feature extraction combined with shallow classifiers, such as Scale-Invariant Feature Transform (SIFT) [12] and Histogram of Oriented Gradients (HOG) [13]. While these approaches achieve high accuracy in scenarios with limited categories, they suffer from insufficient feature representation capability and poor cross-domain adaptability. Breakthroughs in deep learning have led to new frameworks based on Convolutional Neural Networks (CNNs), with classic models including Visual Geometry Group Network (VGGNet) [14], Residual Network (ResNet) [15], and Vision Transformer (ViT) [16]. Although these methods demonstrate strong representational power on large-scale datasets, they face challenges in the current scenario. Specifically, the system only needs to identify anomalies in fully intact particles at the image center, and some anomalies exhibit subtle characteristics.

In the images, only the anomalies in the central particles need to be identified, while the peripheral particles act as noise. Therefore, we adopt the ViT as the backbone network for this study. The self-attention mechanism in ViT enables the network to adjust its focus according to detection requirements, aligning well with our detection objectives. However, this mechanism primarily captures global information and struggles to effectively model contextual relationships between image patches. To address this limitation, we incorporate the Large Selective Kernel (LSK) [17] into the designed dynamic convolutional attention, allowing the network to dynamically adjust its spatial receptive field for better context capture across different objects. Additionally, some anomalies are relatively small, such as minor contamination occlusions, small coating fractures, and small areas of image blur. Since classification networks typically rely on the feature map with the highest downsampling rate and lowest resolution, detecting such small-scale anomalies becomes challenging. To overcome this, we draw inspiration from the Feature Pyramid Network (FPN) [18] and propose the Multi-Scale Feature Fusion Module (MSFFM). MSFFM effectively extracts valuable information from four different hierarchical feature maps in the backbone network, enhancing the network’s ability to detect small anomalies. Furthermore, we integrate the Focal Attention Module (FAM) [19] into MSFFM to help the model focus on critical information, thereby improving detection performance.

The main contributions are summarized as follows.

- A Focal Attention-based Large Convolutional Kernel Network (FA-LCKNet) is proposed for accurately identifying anomalies in particles from images.
- The LSK is introduced into ViT to enable the network to capture both global and local contextual information simultaneously.
- The MSFFM is designed to enhance the detection of small anomalies by effectively leveraging multi-level feature information.
- The FAM is introduced to enhance the network’s focusing capability, enabling it to better attend to regions requiring detection.

The remainder of this paper is organized as follows. Section 2 provides a brief introduction to the background, followed by a detailed description of the network architecture in Section 3. Section 4 presents an evaluation and ablation study based on our self-constructed particle dataset. Finally, Section 5 offers a summary and outlines directions for future work.

## 2. Background

**Figure 1:**
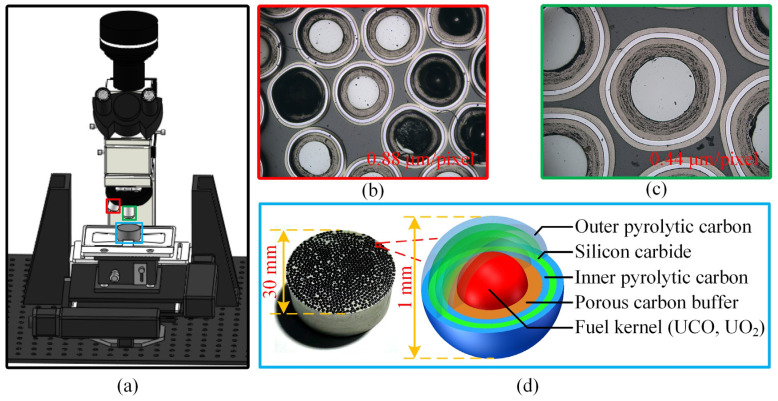
Microscopic imaging device (**a**), image result using 5× objective lens (**b**), and image result using 10× objective lens (**c**) and ceramographic section and coated fuel particles (**d**).

Coated fuel particles serve as the fuel elements for high-temperature gas-cooled reactors (HTGRs), and their quality directly impacts the safe operation of the nuclear reactor. As shown in Figure 1d, at the core of the particle is a uraniferous fuel kernel, surrounded by four coating layers arranged sequentially from the inside out: the porous carbon buffer layer (Buffer), the inner pyrolytic carbon layer (IPyC), the silicon carbide layer (SiC), and the outer pyrolytic carbon layer (OPyC). The thickness of these coating layers significantly affects the stress on the coated fuel particles under irradiation. When the total stress in the coating layers exceeds their fracture strength, the particles may fail [20,21]. Therefore, the coating layer thickness is a critical factor influencing particle performance. Due to manufacturing process variations, discrepancies in coating layer thickness can occur during particle production. As a result, it is necessary to measure the coating layer thickness after particle fabrication. These measurements are used to fine-tune process parameters, thereby enhancing product quality.

Based on this, we have designed a microscopic vision detection system for the rapid measurement of coating thickness. The system comprises a microscopic imaging device, an object detection algorithm, a semantic segmentation algorithm, and other image processing techniques. As shown in Figure 1a, the imaging setup primarily includes a microscope camera, an eyepiece, a light source, 5× and 10× objective lenses, an automatic stage, a lifting device, a controller, and an optical platform. Hundreds of particles were embedded in resin and prepared into a ceramographic section (Figure 1d), which was then placed on the automatic stage for detection. First, the entire sample was scanned under the 5× objective lens (imaging results shown in Figure 1b), and the coordinates of all particles were obtained using object detection. Subsequently, the system switched to the 10× objective lens (imaging results shown in Figure 1c) to detect each particle, and the coating layer thickness was measured using semantic segmentation and image processing techniques.

**Figure 2:**
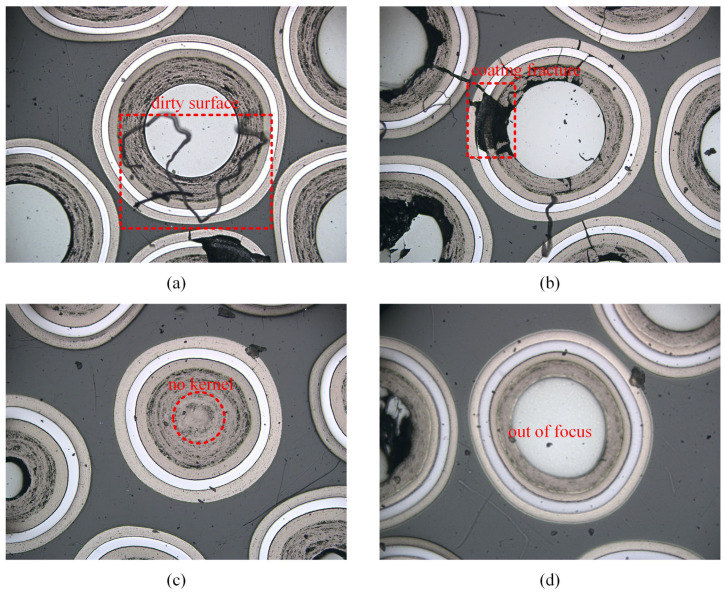
Abnormal situations: dirty surface (**a**), coating fracture (**b**), no kernel (**c**), and out of focus (**d**).

However, during our investigation of semantic segmentation techniques, we found that many of the collected images were either unsuitable for measurement or resulted in biased measurement outcomes. Figure 2 illustrates several anomalies, including surface contamination causing coating occlusion, coating fractures leading to the loss of the target, and missing kernels or image defocus, which can cause measurement data to deviate from the true values. Therefore, it is essential to perform anomaly detection before image analysis, as failing to do so can significantly impact the final measurement accuracy.

## 3. Methodology

### 3.1. Overall Architecture

**Figure 3:**
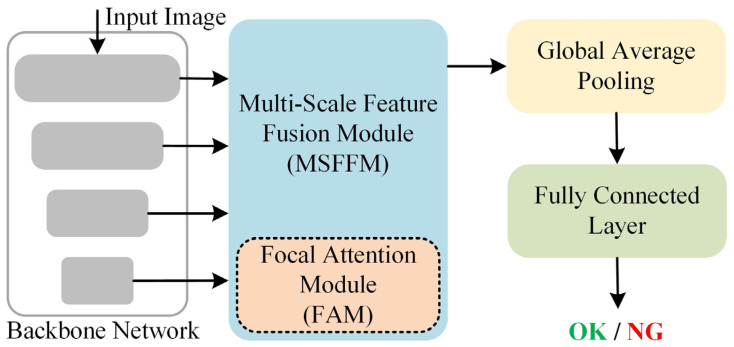
Overview of FA-LCKNet.

The overall architecture of our model is shown in Figure 3. It consists of four main components: the backbone network, the MSFFM (which includes the FAM), Global Average Pooling (GAP), and the Fully Connected Layer. The image is first input into the backbone network (detailed in Section 3.2), where it undergoes encoding through four stages, resulting in four feature maps at different scales. These feature maps are then passed through the MSFFM (detailed in Section 3.3) for multi-scale feature fusion, producing a feature map that integrates multi-level semantic information. The FAM (detailed in Section 3.3), a component of the MSFFM, focuses on the key information in the features, thereby enhancing the model’s recognition ability. Finally, the output is passed through a GAP layer and a Fully Connected Layer to produce the final classification result (OK indicates normal, NG indicates abnormal).

### 3.2. Backbone Network

The ViT successfully adapts the Transformer architecture from natural language processing to computer vision by dividing input images into sequences of 16 × 16-pixel patches (Patch Embedding) and incorporating learnable positional encodings (Positional Embedding) to retain spatial information. It employs a Multi-Head Self-Attention (MHSA) mechanism to model global context, demonstrating the feasibility of a pure Transformer architecture on large-scale datasets such as ImageNet. ViT has shown significant performance advantages over traditional CNNs, particularly in large-scale pretraining scenarios. However, the self-attention mechanism in ViT does not explicitly account for local spatial relationships when computing token associations. This isolated processing approach limits its ability to effectively model the geometric correlations of adjacent pixels, such as edges, textures, and other low-level features.

**Figure 4:**
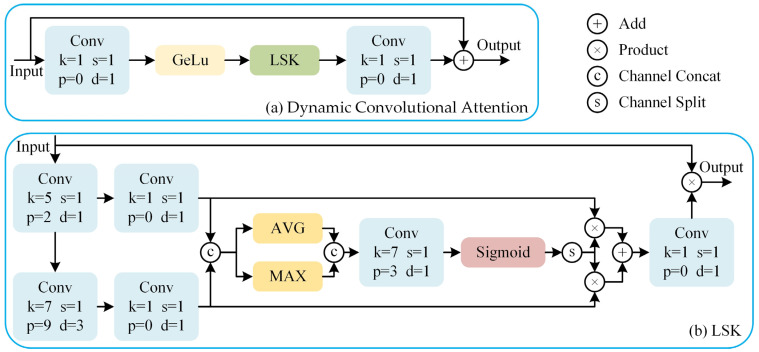
The architecture of the key parts of the backbone network: Dynamic Convolutional Attention (**a**), which replaces the MHSA in the ViT architecture, and the LSK (**b**).

To address this, we introduce the LSK and design a Dynamic Convolutional Attention to replace the MHSA in the ViT architecture, as shown in Figure 4. Unlike the standard ViT, we do not reshape the patch embedding features into 1D token sequences. Instead, we retain their 2D spatial structure and process them directly using convolutional operations. This design choice preserves local spatial relationships, which are critical for capturing fine-grained anomaly patterns, and simplifies the integration of attention mechanisms within a convolutional framework.

The LSK dynamically adjusts the network’s receptive field size according to the requirements. In the LSK, the input features are processed by two different large convolution kernels to capture spatial information at different scales. The outputs of the two convolutions are then concatenated along the channel dimension to allow for the simultaneous utilization of different spatial features in subsequent steps. The concatenated feature map undergoes average pooling and max pooling operations, which are then concatenated along the channel dimension and passed through a convolution to generate a new feature map. This feature map is then passed through a Sigmoid activation function to generate a probability distribution. The probability distribution is element-wise multiplied with the outputs of the two large convolutions to obtain the attention feature map. This step weights the more important regions of the feature map to enhance the network’s ability to recognize specific objects in the image. Finally, the attention feature map is convolved and element-wise multiplied with the input feature map to generate the final output feature map.

### 3.3. Multi-Scale Feature Fusion Module

**Figure 5:**
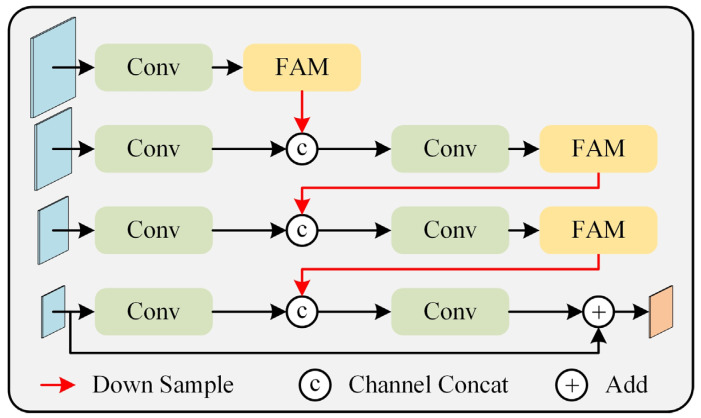
The architecture of MSFFM.

Classification models typically perform subsequent operations on the final feature map of deep convolutional networks. This layer corresponds to a relatively high downsampling rate, such as 16 or 32, which results in less effective information for small objects, leading to poorer recognition performance. To address this issue, common approaches in object detection and semantic segmentation, such as FPN and its variants, are often used. FPN constructs a top-down feature fusion path to merge feature maps at different scales, generating a feature pyramid with rich multi-scale information. Inspired by the concept of FPN, we designed the MSFFM, as shown in Figure 5.

The concept of the MSFFM is similar to FPN; however, it employs a bottom-up feature fusion path. First, convolution operations are performed on the four feature maps at different levels obtained from the backbone network to further extract features. Starting with the lower-level feature map (high resolution), a downsampling operation is applied, followed by concatenation with the higher-level feature map along the channel dimension. The concatenated feature map is then passed through a convolution to reduce the number of feature channels and decrease the computational load of the network. This process is repeated until the highest-level feature map (low resolution) is obtained. The bottom-up feature fusion path, combined with skip connections, helps propagate fine-grained details from lower-level feature maps to higher-resolution feature maps while retaining useful semantic information from different depths. Finally, the obtained feature map is element-wise added to the input feature map at the lowest resolution, resulting in the final output feature map. This residual structure ensures that the network’s performance does not degrade, aiding in model optimization and training.

After the four feature maps of different sizes undergo convolution for feature extraction, they contain a significant amount of high-level semantic information. However, not all of this information is necessarily useful for our detection task. To address this, we designed the FAM, which is a dynamic convolutional attention module that captures key information within the feature maps, allowing the network to focus on the regions that require attention.

**Figure 6:**
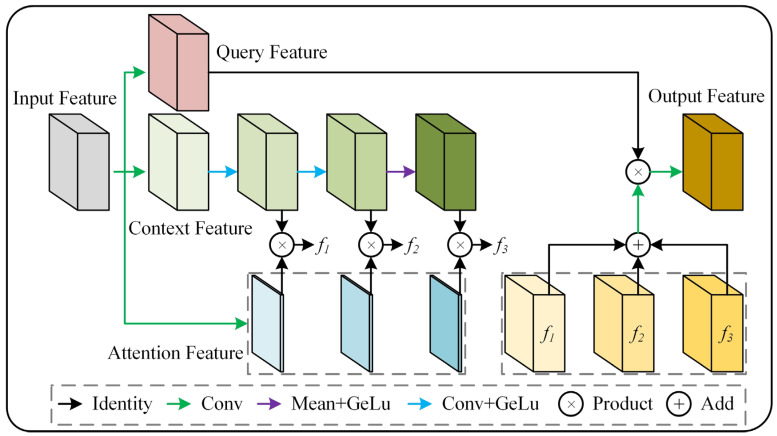
The architecture of FAM.

The architecture of the FAM is shown in Figure 6. This module adopts a multi-branch feature processing mechanism. First, the input feature maps are transformed through parallel depth-wise convolution layers [22] to generate the query features, context features, and attention features, respectively. The context features are processed by a three-stage cascaded encoding unit. The first two stages consist of convolution layers (Conv) followed by GELU activation, while the final stage employs GAP (keep dims) and GELU activation to encode multi-scale features. This progressive encoding strategy incrementally expands the receptive field, enabling hierarchical context extraction from local to global scales. In the feature modulation phase, the output of each encoding stage is element-wise multiplied by the attention weights. The distribution of these weights reflects the degree of correlation between contextual information at different spatial positions and the query features. This dynamic convolutional attention mechanism allows the model to enhance features in specific regions by selectively activating spatial contextual information with high semantic significance. The modulated context features from the three stages are fused through element-wise addition, forming a context representation with multi-scale perception capability. This fusion method preserves the original spatial details while enhancing global semantic consistency through cross-scale feature integration. Finally, the fused context features interact with the query features via element-wise multiplication, followed by a convolution operation to produce the optimized feature representation. This process effectively embeds multi-scale semantic cues into the query feature space, improving the model’s ability to discriminate fine-grained anomalous features.

In traditional self-attention, the query and key features interact to generate weights, which are then used to apply attention to the value features. In FAM, the method of obtaining query features remains unchanged; the core focus is on the extraction and aggregation of contextual information at different granularities. Contextual information is further extracted through three convolutional layers, with the receptive field increasing and the semantic information becoming more advanced as the layers progress, allowing the network to obtain feature maps at different granularities. The three weights determine which information from each layer of contextual features is retained, enabling the network to capture both long-range dependencies and local contextual information after all contextual information is aggregated.

## 4. Experiment and Result Analysis

This study validates the proposed method by constructing a dataset. Section 4.1 details the construction of the dataset and the model training method. Section 4.2 presents the evaluation metrics used to assess the model. Section 4.3 compares the proposed method with several other well-established classification models, including VGGNet-16 [14], ResNet-50 [15], MobileNet-V3 [23], EfficientNet [24], and ViT-Base [16]. Section 4.4 conducts ablation experiments on the proposed improvements to demonstrate their effectiveness.

### 4.1. Dataset Construction and Training Method

#### 4.1.1. Dataset Construction

This study collected 20 sets of ceramic slice samples using a high-resolution microscopic imaging system, resulting in 1400 raw images. The dataset was then randomly divided into training, validation, and test sets in a 7:1.5:1.5 ratio, consisting of 980 training images, 210 validation images, and 210 test images. The training set was used for network parameter optimization, the validation set for hyperparameter tuning during training, and the test set served as the objective basis for evaluating the final model performance.

To enhance data representation capabilities, random geometric transformations were introduced for the training set. With a 50% probability, images were subjected to mirror operations along the horizontal axis, vertical axis, and their combinations, effectively expanding the morphological diversity of the samples. During the data preprocessing phase, samples were categorized into two classes: normal (OK) and abnormal (NG). These labels were manually annotated by domain experts based on whether the surface condition of the particle could adversely affect the measurement of coating layer thickness. Specifically, only samples with sufficient surface integrity to ensure accurate thickness detection were labeled as OK, while those exhibiting defects or contaminants that might interfere with the measurement process were labeled as NG.

Due to hardware limitations, the original high-resolution images (3072 × 4088 pixels) were scaled down proportionally to 768 × 1022 pixels. Specifically, when the ViT architecture was used as the backbone network, non-standard input sizes were expanded to a 1024 × 1024 square format via zero-padding. This approach preserved the aspect ratio of the original images while meeting the technical requirement for regular input tensors in Transformer-based models.

#### 4.1.2. Model Training

To enhance the generalization capability of the model and reduce overfitting, we employed Label Smooth Loss [25] as the classification loss function. Label smoothing is a regularization technique that addresses overconfidence in neural networks by replacing hard one-hot labels with soft targets. Specifically, it introduces noise into the traditional Softmax Loss, enhancing the model’s robustness. In the context of anomaly detection, this mechanism is particularly relevant due to the inherent data imbalance between OK and NG samples. By preventing the model from assigning excessively high probabilities to dominant classes (e.g., OK samples), label smoothing encourages more balanced feature learning, which is critical for detecting rare anomalies.

Assume the original labels are in a one-hot encoding format, where the correct class is denoted as *k*, the total number of classes as *K*, and the smoothing parameter as *ε* (typically set to 0.1 or 0.01). The smoothed label *y_i_* is defined as shown in Equation (1). Essentially, the probability assigned to the correct class is reduced from 1 to (1 − *ε*), while the remaining *ε* is evenly distributed among the other classes. This “softening” of labels acts as an implicit calibration mechanism, reducing model overfitting to training-set noise and improving uncertainty estimation—both essential properties for anomaly detection systems that must handle ambiguous or unseen anomaly patterns.

(1) $$y_{i}=\left\{\begin{array}{ll} 1-\varepsilon & i=k\\ \varepsilon /(K-1) & i\neq k \end{array}\right.$$

The formula for Label Smooth Loss is given in Equation (2), where *p_i_* represents the model’s predicted probability for class *i*. Notably, by penalizing overconfident predictions (e.g., *p_i_*→1) through the smoothed targets, the loss function promotes more conservative decision boundaries. This aligns with the anomaly detection objective of distinguishing subtle deviations from OK patterns rather than merely memorizing training data distributions.

(2) $$Loss=-\sum_{i=1}^{K}y_{i}\log(p_{i})$$

The hardware, software, and environmental configurations used in this study include an Intel i5-11400F processor (Intel, procured in China), an NVIDIA GeForce GTX 3060 GPU (NVIDIA, procured in China), CUDA 12.1, cuDNN 8.9.7, Python 3.11.9, PyTorch 2.3.1, and MMPretrain 1.0.1 [26]. These configurations provide the necessary computational power for efficient model training. Additionally, we carefully optimized hyperparameters such as the learning rate, batch size, and number of epochs to achieve optimal performance. A summary of these settings is provided in Table 1.

**Figure 7:**
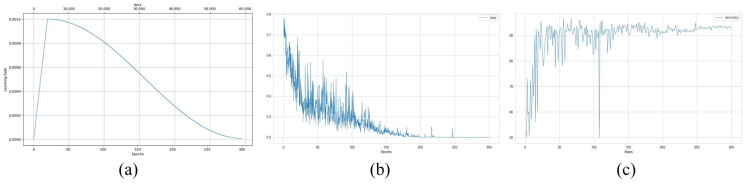
Parameter variation curves during network training: learning rate (**a**), loss (**b**), accuracy (**c**).

To validate the effectiveness of the training strategy, we conducted a visual analysis of key hyperparameter trends, as illustrated in Figure 7. During training, we employed a learning rate warmup strategy [28], where the learning rate was linearly increased over the first 20 epochs. This approach effectively mitigates initial oscillations caused by abrupt parameter updates, allowing the network to reach a stable convergence phase more efficiently. In the later stages, we adopted the Cosine Annealing Learning Rate strategy [29], which gradually decays the learning rate following a half-period cosine function. This design ensures a balance between continued exploration in the parameter space and fine-tuned optimization of network weights.

### 4.2. Evaluation Metrics and Evaluation Results

We employ various metrics to evaluate the performance of the proposed network model in particle anomaly detection, including Precision, Recall, F1-Score, and Frames Per Second (FPS). Precision measures the false positive rate for each category, as defined in Equation (3). Recall assesses the false negative rate for each category, as defined in Equation (4). F1-Score represents the weighted average of Precision and Recall, providing a comprehensive measure of the model’s overall performance, as shown in Equation (5). FPS evaluates the detection efficiency of the model.

(3) Precision=TP/(TP+FP)

(4) Recall=TP/(TP+FN)

(5) F1-Score=2×(Precision×Recall)/(Precision+Recall)

where True Positive (TP), False Positive (FP), and False Negative (FN) are the number of truly predicted positive instances, false positive instances, and false negative instances, respectively.

A comprehensive evaluation demonstrates that the proposed model achieves outstanding performance in particle anomaly detection (detailed in Table 2). For anomalous sample identification, the model achieves a Precision of 98.04% (NG category), indicating high accuracy in anomaly discrimination and effective mitigation of false alarms. Meanwhile, the Recall for OK samples reaches 98.10%, ensuring the system captures potential anomalies with minimal missed detections.

Notably, the Recall for NG samples (95.24%) and the Precision for OK samples (95.37%) exhibit room for optimization. The lower NG-Recall suggests that a small number of OK samples may remain undetected, while the OK-Precision indicates occasional misclassification of OK samples as NG. Further analysis, considering the practical metallographic inspection scenario—where thousands of particles exist, but only 200 require actual detection—reveals that these minor misclassifications do not substantially impact the final results. Experimental data further validates the model’s effectiveness in anomaly detection. While its processing speed of 22.4 FPS is slower than some lightweight models, it fully meets the real-time processing requirements of industrial inspection applications.

**Figure 8:**
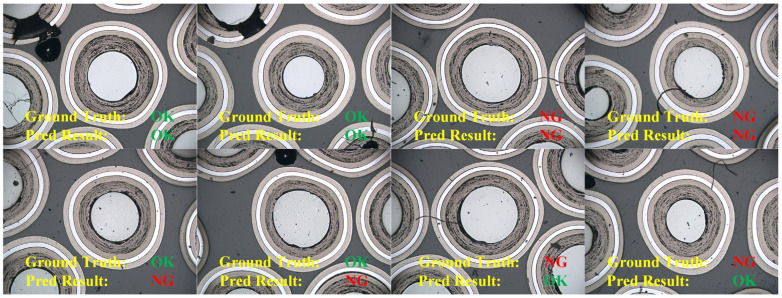
Representative examples of correct detection results (**first row**) and incorrect detection results (**second row**) are shown.

To provide a more intuitive understanding of the proposed method’s performance, Figure 8 presents visualized examples of detection results, including both successful and failed cases. As shown in the first row of images, the model correctly classifies both OK and NG samples. These successful cases demonstrate that the model is not affected by edge particle damage in OK samples and can effectively focus on subtle anomalies in NG samples to achieve accurate classification. In contrast, the second row highlights failure cases. Although the difficulty of detecting anomalies appears similar between the two rows based on visual inspection, the model makes incorrect predictions for some images, making it challenging to identify the underlying causes. To further investigate this issue, we visualized the output feature maps from the final FAM in an attempt to uncover possible reasons for the misclassification.

**Figure 9:**
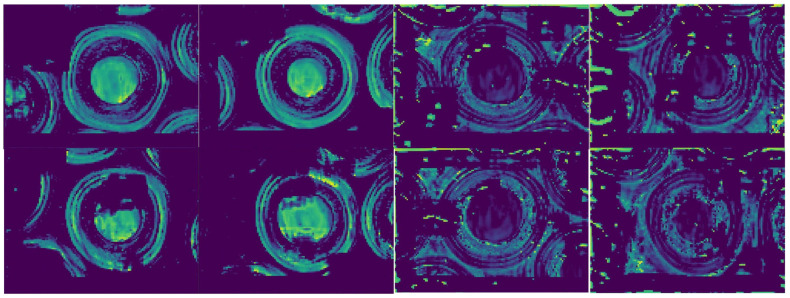
Visualization of the output features from the final FAM corresponding to each example image in Figure 8.

The visualization of the output feature maps from the final FAM is shown in Figure 9. For consistency, the image layout follows the same order as in Figure 8. From the four OK samples on the left, it can be observed that the model effectively suppresses irrelevant disturbances, such as edge particle damage. The feature responses are primarily concentrated in the central particle regions of the images. However, it is noteworthy that two of these samples were misclassified as NG—indeed, they are the only misclassified OK samples. We suspect this may be due to the use of Label Smoothing Loss. Although label smoothing serves as an effective regularization technique, it may introduce noise during training, particularly under class-imbalanced conditions where OK samples dominate. This may inadvertently bias the model against OK samples near the decision boundary. For the four NG samples on the right, most of the feature maps exhibit clear responses in the anomalous regions. However, in the bottom-right image, the anomaly response appears relatively weak, which may have led to a false-negative prediction. More intriguingly, in the remaining three images, despite strong activations in the anomalous regions, the model still misclassifies one of them as OK. This suggests that while the model can detect fine and subtle contaminants, it may still struggle to confidently classify anomalies that lie near the OK/NG decision boundary—particularly when the anomalies are extremely subtle.

**Figure 10:**
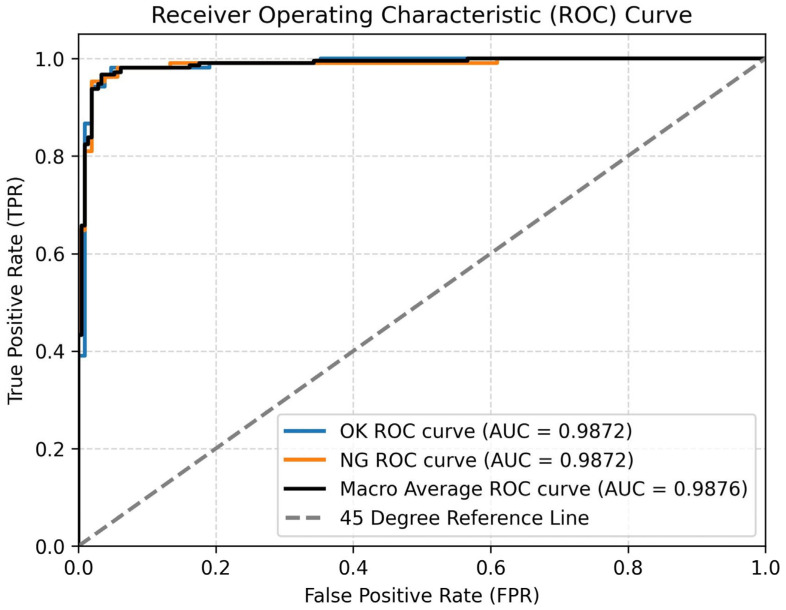
ROC Curve.

To provide a more intuitive and objective assessment of the model’s classification performance, the Receiver Operating Characteristic (ROC) curves are presented in Figure 10. These curves plot the True Positive Rate (TPR) against the False Positive Rate (FPR) across a range of classification thresholds, offering insights into the trade-off between sensitivity and specificity. As shown in Figure 8, both the OK and NG classes achieve high Area Under the Curve (AUC) scores of 0.9872, with a macro-average AUC of 0.9876, indicating that the model performs robustly and consistently across both classes. The high AUC values suggest excellent separability between OK and NG samples, enabling the model to make confident and reliable predictions. Notably, the ROC curves for the OK and NG classes nearly overlap, demonstrating the model’s balanced classification ability without significant bias toward either class. This further validates the effectiveness of the proposed feature aggregation and attention mechanisms in capturing subtle distinctions between OK and NG instances.

### 4.3. Comparison Studies

We compare our proposed approach against several representative deep learning-based models, including VGGNet-16, ResNet-50, MobileNet-V3, EfficientNet, and ViT-Base. These models have demonstrated strong generalization capabilities across various visual tasks. VGGNet-16 serves as a classical chain-structured CNN baseline to verify fundamental feature extraction capabilities. ResNet-50, by introducing residual connections, overcomes limitations in network depth and represents a key direction in deep network optimization. MobileNet-V3 and EfficientNet reflect two distinct paradigms of efficiency enhancement: lightweight design for mobile deployment and compound scaling for server-side performance, respectively. ViT-Base exemplifies the groundbreaking application of Transformer architectures in the vision domain. Collectively, these models establish a comprehensive and multi-dimensional comparison framework, spanning conventional CNNs, modern lightweight architectures, and attention-based mechanisms. The selection includes widely adopted industrial benchmarks as well as state-of-the-art innovations from recent years.

We conducted a comprehensive comparative study to systematically evaluate our detection framework against state-of-the-art methods in the field. The primary objective of this rigorous comparison was to quantify the competitive advantage in detection accuracy, specifically in terms of Precision, Recall, and F1-Score. As summarized quantitatively in Table 3, our method significantly outperforms existing approaches, achieving a Precision of 96.71% and a Recall of 96.67% while maintaining a competitive balance between Precision and Recall.

A systematic analysis of the experimental data in Table 3 reveals significant performance differences among various models in the anomaly detection task. Specifically, VGGNet-16 underperforms in two key metrics: NG-Precision (83.05%) and OK-Recall (80.95%), likely due to inherent limitations of its stacked small-kernel convolutional architecture, which restricts the receptive field and hampers global context modeling. ResNet-50, leveraging residual connections and deep convolutional stacking, achieves substantial improvements over VGGNet-16, with increases of 9.18% in NG-Precision and 11.43% in OK-Recall. Meanwhile, the lightweight MobileNet-V3 exhibits an excellent balance between accuracy and efficiency, maintaining detection performance comparable to ResNet-50 while achieving the highest FPS (144.6)—primarily due to its inverted residual structure and search-optimized channel compression strategy. EfficientNet, which scales depth, width, and resolution using a unified compound coefficient, delivers a well-balanced performance improvement. ViT-Base, leveraging self-attention mechanisms for global context modeling, surpasses EfficientNet in NG-Precision and OK-Recall by 1.69% and 1.9%, respectively. However, its limited local feature extraction capability constrains its improvement in fine-grained anomaly detection. The proposed method achieves the best results in F1-Score (96.67%), NG-Precision (98.04%), and OK-Recall (98.10%), although it records the lowest FPS (22.4). However, in practical applications, this speed remains sufficient, indicating that our method effectively balances detection accuracy and efficiency.

**Figure 11:**
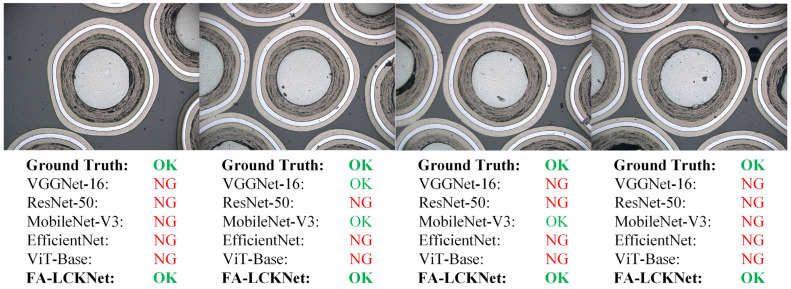
Detection results on OK samples by different models.

**Figure 12:**
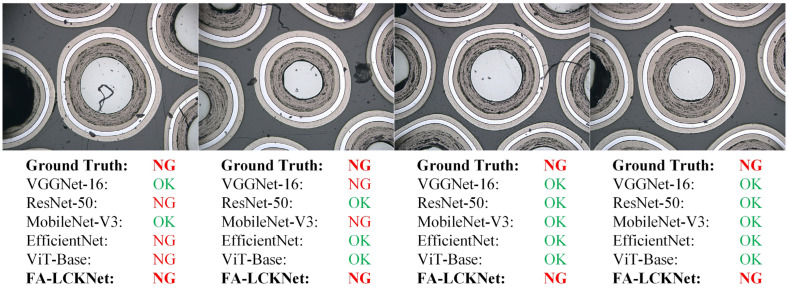
Detection results on NG samples by different models.

To provide a more intuitive comparison of model performance in practical detection scenarios, Figure 11 and Figure 12 illustrate the detection results of four representative OK samples and four representative NG samples, respectively, across different models. As shown in Figure 11, although the OK samples are free from edge particle damage, several competing models misclassify multiple instances, whereas the proposed model correctly identifies all as OK. Similarly, in the NG samples shown in Figure 12, our model exhibits superior detection capability, successfully identifying all samples with subtle anomalies. In contrast, the other models demonstrate inconsistent performance in recognizing these defects, with some failing to capture localized abnormal regions, thereby highlighting their limitations in anomaly representation. These visual results indicate that the proposed model offers stronger discriminative ability for particle anomaly detection.

### 4.4. Ablative Studies

Systematic ablation studies were conducted to quantitatively assess the effectiveness of the proposed architectural improvements. Subsequently, three components (LSK, MSFFM, and FAM) were incrementally integrated into the baseline architecture. As shown in Table 4, this stepwise integration reveals several key findings. First, incorporating the LSK module led to an average improvement of approximately 2 percentage points across all performance metrics. Second, integrating the MSFFM module resulted in no significant changes in the non-critical metrics OK-Precision and NG-Recall, whereas the critical metrics NG-Precision and OK-Recall both improved by approximately 2%. By employing a cross-scale feature fusion strategy, this module adaptively weights fine-grained high-resolution features and deep semantic representations, significantly enhancing the detection of subtle anomalies. Finally, after introducing the FAM module, non-critical metrics exhibited a stable improvement of about 2%, while critical metrics remained consistent. This module enables the model to focus more effectively on central particle regions, mitigating interference from edge particles and reducing false positives and false negatives for OK samples. The experimental results demonstrate that the three modules work synergistically to achieve a comprehensive performance enhancement, validating the hierarchical and complementary nature of the module design.

In addition, further analysis of Table 4 reveals that, under the integration of MSFFM and FAM, the overall performance of the model is further enhanced with the introduction of LSK, thereby validating the effectiveness of the LSK enhancement. Notably, the inclusion of FAM significantly improves the NG-Recall and the OK-Precision, while the NG-Precision and the OK-Recall remain largely unchanged or only slightly improved. This observation can be attributed to the design of FAM, which aims to enhance contextual awareness and multi-scale feature integration. Specifically, FAM aggregates visual context from different receptive fields and selectively emphasizes discriminative regions, which is beneficial for detecting subtle or spatially dispersed anomalies. As a result, the model becomes more sensitive to true NG instances, thereby improving NG-Recall. At the same time, by suppressing irrelevant or noisy activations in the feature space, FAM enables the model to classify OK samples with greater confidence, contributing to improved OK-Precision. However, since FAM is optimized for capturing complex and diverse NG patterns, it may slightly increase the false positive rate in NG predictions, which explains the limited gain in NG-Precision. Similarly, OK-Recall is constrained by the model’s ability to fully capture intra-class variability within OK samples, which is less influenced by FAM’s focus on NG contextual cues. In summary, FAM enhances the model’s discriminative capacity for NG and increases confidence in OK predictions, but its benefits are primarily reflected in improved Recall for challenging NG samples rather than across all performance metrics.

## 5. Conclusions

This study addresses the technical challenges in coated fuel particle anomaly detection by proposing a FA-LCKNet. To tackle the dual challenges of interference from peripheral particles in central particle detection and the difficulty in identifying subtle anomalies, we integrate a ViT backbone with an LSK module, constructing a multi-scale representation framework that enhances both global perception and local feature extraction. The MSFFM is specifically designed to establish inter-feature interactions, significantly improving the detection rate of minor anomalies. Additionally, the FAM introduces dynamic weight allocation within the detection regions, enabling the network to effectively suppress background noise while focusing on critical features. Experimental results show that our method achieves a 1.96% false detection rate and 1.9% miss detection rate in particle anomaly detection on the test set, which significantly outperforms baseline models such as VGGNet, ResNet, MobileNetV3, EfficientNet, and ViT. While there remains room for optimization in computational efficiency, the proposed approach meets the requirements for practical engineering applications. This research provides a robust solution for evaluating the integrity of nuclear fuel particle coatings and lays a solid foundation for precise coating thickness measurement in future studies.

Although this study has made progress in detecting anomalies in coated fuel particles, several areas require further improvement. (1) The current model still has limitations in identifying subtle anomalies. Future work will incorporate multimodal feature alignment techniques to achieve a breakthrough in reducing the overall error rate to ≤1%. (2) To meet real-time requirements in industrial applications, a lightweight network architecture based on knowledge distillation will be developed to improve inference speed while maintaining detection accuracy. (3) Cross-domain adaptation strategies will be explored to enhance the model’s robustness across different particle configurations.

## Figures and Tables

**Table 1:** The hyperparameter settings.

Item	Value
Batch Size	4
Epochs	300
Optimizer	AdamW [27]
Learning Rate	1 × 10^−3^
Weight Decay	0.05
Eps	1 × 10^−8^
Betas	0.9, 0.999
Warmup Type	LinearLR
Warmup Epochs	20
Warmup Start Factor	1 × 10^−3^
Learning Rate Scheduler	CosineAnnealingLR
Minimum Learning Rate	1 × 10^−5^

**Table 2:** Model evaluation results.

Class	Precision	Recall	F1-Score	FPS
OK	95.37%	98.10%	96.71%	22.4
NG	98.04%	95.24%	96.62%	
AVG	96.71%	96.67%	96.67%	

**Table 3:** Effects of different models (bold values indicate the best performance in each column).

Model	Precision (%)			Recall (%)			F1-Score (%)			FPS
	OK	NG	AVG	OK	NG	AVG	OK	NG	AVG	
VGGNet-16	92.40	83.05	87.73	80.95	93.33	87.14	86.29	87.89	87.09	24.9
ResNet-50	90.65	92.23	91.44	92.38	90.48	91.43	91.51	91.35	91.43	28.4
MobileNet-V3	90.57	91.35	90.96	91.43	90.48	90.96	91.00	90.91	90.96	**144.6**
EfficientNet	92.31	91.51	91.91	91.43	92.38	91.91	91.87	91.94	91.91	40.4
ViT-Base	91.59	93.20	92.40	93.33	91.43	92.38	92.45	92.31	92.38	23.0
**Ours**	**95.37**	**98.04**	**96.71**	**98.10**	**95.24**	**96.67**	**96.71**	**96.62**	**96.67**	22.4

**Table 4:** Effects of different modules (bold values indicate the best performance in each column).

Module			Precision (%)			Recall (%)			F1-Score (%)		
LSK	MSFFM	FAM	OK	NG	AVG	OK	NG	AVG	OK	NG	AVG
			91.59	93.20	92.40	93.33	91.43	92.38	92.45	92.31	92.38
✓			93.52	96.08	94.80	96.20	93.33	94.77	94.84	94.69	94.77
✓	✓		93.64	98.00	95.82	**98.10**	93.33	95.72	95.81	95.61	95.71
	✓	✓	93.99	95.96	94.98	96.19	93.47	94.83	95.08	94.70	94.89
✓	✓	✓	**95.37**	**98.04**	**96.71**	**98.10**	**95.24**	**96.67**	**96.71**	**96.62**	**96.67**

## Data Availability

The datasets presented in this article are not readily available because they are subject to confidentiality agreements and institutional data protection policies.
